# Unbalance Between Sarcoplasmic Reticulum Ca^2 +^ Uptake and Release: A First Step Toward Ca^2 +^ Triggered Arrhythmias and Cardiac Damage

**DOI:** 10.3389/fphys.2019.01630

**Published:** 2020-01-23

**Authors:** Marilén Federico, Carlos A. Valverde, Alicia Mattiazzi, Julieta Palomeque

**Affiliations:** ^1^Centro de Investigaciones Cardiovasculares “Dr. Horacio E. Cingolani”, CCT-La Plata/CONICET, Facultad de Cs. Médicas, Universidad Nacional de La Plata, La Plata, Argentina; ^2^Centro de Altos Estudios en Ciencias Humanas y de la Salud, Universidad Abierta Interamericana, Buenos Aires, Argentina

**Keywords:** sarcoplasmic reticulum Ca^2+^-ATPase, ryanodine receptor 2, arrhythimas, phospholamban, apoptosis, diabetic myocardiopathy, isquemia-reperfusión

## Abstract

The present review focusses on the regulation and interplay of cardiac SR Ca^2+^ handling proteins involved in SR Ca^2+^ uptake and release, i.e., SERCa2/PLN and RyR2. Both RyR2 and SERCA2a/PLN are highly regulated by post-translational modifications and/or different partners’ proteins. These control mechanisms guarantee a precise equilibrium between SR Ca^2+^ reuptake and release. The review then discusses how disruption of this balance alters SR Ca^2+^ handling and may constitute a first step toward cardiac damage and malignant arrhythmias. In the last part of the review, this concept is exemplified in different cardiac diseases, like prediabetic and diabetic cardiomyopathy, digitalis intoxication and ischemia-reperfusion injury.

## Introduction

Cardiovascular diseases are the leading cause of morbidity and mortality worldwide, being Ca^2+^ mishandling one of the most striking abnormalities in the setting of a wide spectrum of pathologies, including cardiac hypertrophy, heart failure, DCM, and I/R damage. Indeed, alterations that constitute the hallmark of these diseases, like contractile dysfunction, cardiac arrhythmias or cell death are in great part a reflection of the impairment in Ca^2+^ handling and the altered function of the SR, a pivotal responsible of Ca^2+^ cycling within cardiac myocytes.

The purpose of this review is: (1) To summarize the regulation of the main cardiac SR Ca^2+^ handling proteins involved in SR Ca^2+^ uptake and release, i.e., SERCA2a/PLN and RyR2. Both proteins are highly regulated by additional partners’ proteins and/or PTMs that may increase or decrease their activity. (2) To describe how the disruption of the interplay among these proteins may constitute a key determinant of Ca^2+^ triggered arrhythmias and cardiac damage. (3) To recapitulate experimental evidence in which alterations of the balance between these key players contribute to SR Ca^2+^ mishandling with the ensuing production of Ca^2+^ triggered arrhythmias and cell death in different cardiac diseases.

## Excitation-Contraction Coupling

**FIGURE 1:**
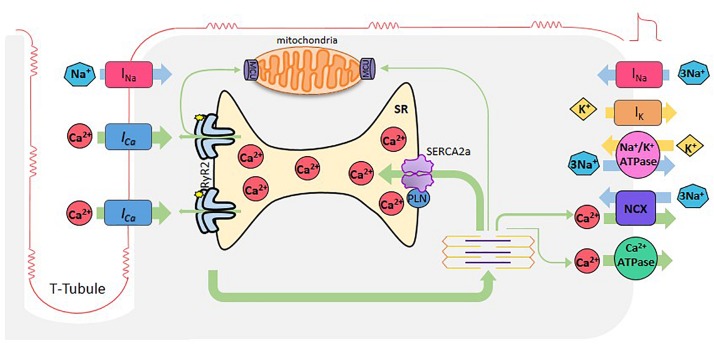
Cardiac excitation-contraction coupling. When cardiomyocytes are reached by an AP, depolarization of the plasma membrane by Na^+^ entry (I_Na_), induces the opening of L-type Ca^2+^ channels. Ca^2+^ entering through these channels (Ca^2+^ current, I_Ca_), induces Ca^2+^ release by the RyR2s (Ca^2+^-induced- Ca^2+^-release), which subsequently activates myofilaments for muscle contraction. Relaxation occurs when SR -Ca^2+^ ATPase (SERCA2a) reuptakes Ca^2+^, lowering cytosolic Ca^2+^ concentration in combination with Ca^2+^ extrusion via the Na^+^- Ca^2+^ exchanger (NCX) working in reverse mode and the sarcolemma Ca^2+^-ATPase. Mitochondria also participate taking and extruding Ca^2+^ from the cytosol during Ca^2+^ cycle. Green arrows depict Ca^2+^ fluxes.

Cardiomyocyte excitation-contraction coupling (ECC) is the process that links membrane depolarization, at the surface cell level, with myofilament interaction that drives contraction, inside the cell. Ca^2+^ ions are the link between the two processes (Figure 1).

## Regulation of Ca^2+^ Handling Proteins Involved in Sr Ca^2+^ Uptake and Release

The SR orchestrates the ECC, being Ca^2+^ ions the main players. Ryanodine Receptors 2 (RyR2) and SERCA2a are highly and precisely regulated by several proteins and kinases, which allow a fine-tuned synchronization of Ca^2+^ cycling and therefore of cardiac contraction and relaxation processes. We will mention here several key modulators and PTMs of these two main proteins. For a further review see for instance (Kranias and Hajjar, 2012; Haghighi et al., 2014; Mattiazzi and Kranias, 2014; Eisner et al., 2017; Meissner, 2017; Santulli et al., 2017a, b).

### Regulation of SR Ca^2+^ Uptake

#### SERCa2a Post-translational Modifications

Redox regulations appear to play an important role in SERCA2a function, both in health and disease. In cardiac myocytes it has been shown that oxidative stress reduces contractility with depletion of SR Ca^2+^ stores, due to SERCA2a inhibition (Morris and Sulakhe, 1997; Xu et al., 1997; Kaplan et al., 2003; Kuster et al., 2010). Indeed, several pathologies like metabolic syndrome (Balderas-Villalobos et al., 2013) or atherosclerosis (Cohen and Adachi, 2006), which are associated with an increase in oxidative stress, reduced SERCA2a activity and contractility.

Nitric oxide activates SERCA2a activity by a cGMP-independent pathway which involves the direct modification of reactive thiol groups on the protein, not only of vascular smooth muscle but also of cardiac and skeletal muscle. On its own, NO is a weak SH-group oxidant. However, in the presence of O_2_^–^ it results in an increased ONOO^–^ production. Under physiological conditions, the ONOO^–^ produced may react with the thiol groups of proteins producing S-nitrosylation and S-gluthationylation of cysteine residues. S-gluthationylation increases the activity of the pump. The SERCA2a residue mainly involved in this reaction is the reactive thiol group Cys^674^ (Adachi et al., 2004). However, an exacerbated increase of ONOO^–^ nitrosylates the hydroxyl groups of SERCA2a, producing impairment of cardiac relaxation (Braun et al., 2019).

Sarcoplasmic reticulum Ca^2+^-ATPase is also regulated by the SUMO1 (sumoylation), by AGEs (glycation) and by acetylation/deacetylation processes. SERCA2a sumoylation appears to prolong the lifetime of SERCA2a as well as to increase its intrinsic activity by SUMO1 binding to Lys480 and Lys585 residues. Indeed, increasing SUMO1 expression restores SERCA2a levels, improves hemodynamic performance, and reduces mortality in heart failure (Kho et al., 2011). AGEs complexes can compromise the pump activity by altering the structural movements required for translocating Ca^2+^ from the cytosol to the lumen of the SR (Bidasee et al., 2004). Finally, recent experiments indicated that the acetylation of SERCA2a at K492 site was significantly increased in heart failure (HF) in association with a reduction of SIRT1, a class III histone deacetylase. Acetylation of K492 significantly diminished SERCA2a activity, possibly by interfering with the binding of ATP. Activation of SIRT1 restored SERCA2a activity. This strategy may, therefore, be useful for HF treatment (Gorski et al., 2019).

#### PLN Post-translational Modifications and the PLN Interactome

**FIGURE 2:**
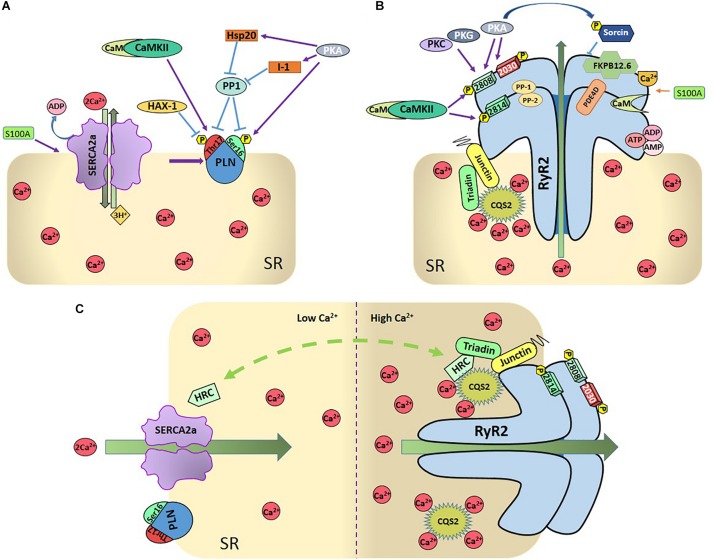
**(A)** Regulation of SR-Ca^2+^ uptake. SR- Ca^2+^ uptake takes place through the SR- Ca^2+^ ATPase (SERCA2a), being PLN the main regulatory protein of SERCA2a. PLN can be phosphorylated by PKA at Ser^16^ residue and by Ca^2+^-calmodulin-dependent protein kinase (CaMKII) at Thr^17^ site. Protein phosphatase 1 (PP1) dephosphorylates both sites. Either one or both phosphorylation sites relieve PLN inhibition on SERCA2a. Several other proteins regulate SERCA2 either directly, like S100A or Sumo1, or indirectly, through the regulation of PLN (like the hematopoietic lineage cell-specific protein-1 (HS-1) associated protein X-1 (Hax-1), the Heat-shot protein 20 (Hsp20), and the inhibitor 1 (I-1). As described in the text, PLN and SERCA2a are also regulated by redox processes and SERCA by acetylation and glycation (Not shown in the Figure for the sake of clarity). **(B)** Regulation of SR-Ca^2+^ release: SR-Ca^2+^ release occurs mainly through the RyR2 which are also highly regulated. CaMKII phosphorylates RyR2 at Ser2814 and S2808 sites and PKA at Ser2808 and 2030 sites. Ser2808 is also target of protein-kinase C (PKC) and protein-kinase G (PKG). PP1 and PP2 are the phosphatases that dephosphorylate RyR2. Other regulatory proteins are sorcin, S-100 at the cytosolic side, FKB12.6 and calmodulin (Cm), bounded to RyR2, CASQ2, triadin, and juntin at the cytosolic site. Phosphodiesterase (PDE), ions, and nucleoside phosphates (AMP, ADP, and ATP) are also bounded to RyR2. **(C)** Dual role of HRC: The intra-luminal protein histidine-rich calcium binding protein (HRC) interacts with SERCA2a as well as with triadin in a Ca^2+^-dependent fashion, increasing RyR2 Ca^2+^ release and SERCA2a Ca^2+^-uptake when Ca^2+^ increases in SR.

Undoubtedly, the main regulator of SERCA2a activity is PLN (Tada et al., 1975). PLN is a small protein (52 amino acid residues) that binds to and allosterically inhibits SERCA2a (MacLennan and Kranias, 2003). Dephosphorylated PLN reduces the affinity of SERCA2a for Ca^2+^ whereas PLN phosphorylation increases SERCA2a pump activity. There are two PLN phosphorylation sites that are physiologically relevant: Ser^16^ residue, phosphorylated by PKA and Thr^17^ site, phosphorylated by the Ca^2+^-calmodulin-dependent protein kinase II (CaMKII) (Figure 2A). Phosphorylation of these sites increases the affinity of SERCA2a for Ca^2+^ and the rate of SR Ca^2+^ uptake. This, in turn, leads to increases in SR Ca^2+^ load, SR Ca^2+^ release and myocardial contractility (Lindemann et al., 1983; Lindemann and Watanabe, 1985; Mundina de Weilenmann et al., 1987; Mundina-Weilenmann et al., 1996).

The status of PLN phosphorylation, as is the case of any other protein, depends on the dynamic balance between the activity of kinases and phosphatases that phosphorylate and dephosphorylate the protein, respectively. PP1, is the major SR phosphatase that specifically dephosphorylates PLN (Steenaart et al., 1992). Inhibition of PP1 results in increased phosphorylation of PLN and SERCA2a activation (Haghighi et al., 2015; Figure 2A). During β-ARS, PKA phosphorylates PLN at Ser^16^ site and simultaneously inhibits PP1 through the PKA dependent phosphorylation of two additional proteins, inhibitor-1 (I-1) and the small heat shock protein 20 (Hsp20) (Qian et al., 2011). Under β-ARS, PLN-Thr^17^ site is also phosphorylated by CaMKII activation due to the increase in intracellular Ca^2+^ and the inhibition of PP1, produced by the activity of PKA. In contrast, phosphorylation of Thr^17^ of PLN does not occur when only intracellular Ca^2+^ was increased without PKA activation, which is necessary to inhibit PP1 (Mundina-Weilenmann et al., 1996).

Post-translational modification of phospholamban by reactive oxygen and nitrogen species (ROS and RNS, respectively) may also influence SR Ca^2+^ uptake (Bigelow and Squier, 2005; Froehlich et al., 2008; Lancel et al., 2009; Ha et al., 2011). Among these PTM it is interesting to mention the one-electron reduction product of NO, nitroxyl (HNO). This molecule has received special attention not only as a possible signaling molecule in the cardiovascular system but also as a potential therapeutic strategy for HF treatment due to its positive inotropic and lusitropic effects in normal and failing canine hearts (Paolocci et al., 2001, 2003; Sivakumaran et al., 2013). PLN played a central role in these effects of HNO, by enhancing SERCA2a activity (Froehlich et al., 2008). Moreover, it has been suggested that S-nitrosylation of PLN at Cys^36^ and Cys^41^ modulates the PLN-dependent regulation of SERCA2a during β-ARS, i.e., S-nitrosylation of PLN is required for stabilization of the pentameric form of PLN, and consequent SERCA2a activation (Irie et al., 2015). Several additional regulatory proteins are associated with PLN and SERCA2a and contribute to the control of SR Ca^2+^-transport. These include the hematopoietic lineage cell-specific protein-1 (HS-1) associated protein X-1 (HAX1), a ∼35 kDa protein, which was identified forming a complex with HS-1 in lymphocytes (Suzuki et al., 1997), the intra-luminal histidine-rich Ca^2+^ binding protein (HRC), which has been shown to interact with both SERCA2a and triadin on the SR luminal side (see below and Figure 2C) and S100A1 on the cytosolic side (Kiewitz et al., 2003). Ca^2+^-dependent S100A1 binding to SERCA2a results in an increased enzymatic activity which is associated with enhanced SR Ca^2+^ uptake and load (Most et al., 2001; Kiewitz et al., 2003; Kettlewell et al., 2005). As will be discussed below, HRC and S100A1 also interact and regulate RyR2 and SR Ca^2+^ release (Völkers et al., 2007; for review, see Haghighi et al., 2014; Kranias and Hajjar, 2017; Arvanitis et al., 2018).

### Regulation of SR Ca^2+^ Release

Ryanodine receptor 2 is the largest ion channel known in nature and one of the most relevant Ca^2+^ handling proteins. RyR forms a homotetrameric assembly comprising four monomers of 565 kDa each (Van Petegem, 2015). There are three mammalian isoforms that share 65% sequence identity: RyR1, predominantly expressed in skeletal muscle; RyR2, the cardiac isoform and RyR3, expressed in several tissues including the brain (Capes et al., 2011). While at the cytosolic portion, the channel contains multiple regulatory domains, such as binding sites for energy sensors (ATP, ADP, and AMP) (Figure 2B), and inorganic phosphate, metabolites such as pyruvate, fatty acids and polyamines, and ions (Mg^2+^, H^+^, and Cl^–^, not shown in the Figure for the sake of clarity) (Zucchi and Ronca-Testoni, 1997; Fill and Copello, 2002; Meissner, 2004); the Ca^2+^ binding site is located in the core domain of the channel just above the transmembrane domain an involves de carboxyl-terminal domain region (Murayama et al., 2018). This complex is also regulated and modulated by a diverse array of RyR2-interacting proteins which involve PKA, CaMKII, phosphatases (i.e., phosphatase 1 and 2A), and phosphodiesterase (PDE4D) which are tethered to the channel and held near their target sites by means of anchoring proteins (Marks, 2002; Lehnart et al., 2005). This allows for a tight and spatially confined homeostatic regulation of the balance between RyR2 phosphorylation and phosphatase dependent dephosphorylation.

#### Post-translational Modifications of RyR2

Phosphorylation is possibly the most studied and controversial PTM modification of RyR2. Phosphorylation of the channel modulates the effect of Ca^2+^ on the RyR2 without having the inherent ability to open or close the channel *per se* (Camors and Valdivia, 2014). Until now three phosphorylation sites in the RyR2 have been identified: Ser^2808^, Ser^2814^, and Ser^2030^ (Figure 2B). Serine 2808 (Ser^2808^, mouse, and Ser2809 in human and canine RyR2 nomenclature) was first described by Witcher and collaborators as a CaMKII site (Witcher et al., 1991). Further in-depth studies of this phospho-site indicated that Ser^2808^ is a target for PKA, CaMKII and possibly PKG (Jiang et al., 2002; Rodriguez et al., 2003; Stange et al., 2003; Currie et al., 2004; Ai et al., 2005; Xiao et al., 2005; Carter et al., 2006; Kohlhaas et al., 2006; Ferrero et al., 2007; Huke and Bers, 2008; MacDonnell et al., 2008; Fischer et al., 2013). Experiments by Marx et al. (2000) indicated that PKA-dependent phosphorylation of RyR2 at Ser^2808^ site under β-ARS, increases P_0_ and SR Ca^2+^ release. However, this contention was not supported by different studies and the functional meaning of this phosphorylation is not clear yet (Xiao et al., 2006; Ferrero et al., 2007; Huke and Bers, 2008). This is in part due to the fact that most studies found that Ser^2808^ is constitutively phosphorylated under basal conditions (Jiang et al., 2002; Rodriguez et al., 2003; Carter et al., 2006; Ferrero et al., 2007; Huke and Bers, 2008), generating doubts about the relevance of “extra” phosphorylation on this site. Moreover, it was also showed that RyR2s were hyperphosphorylated in failing hearts from humans and dogs at Ser^2809^, which was attributed at least in part to a decrease in the amount of PP1 associated to RyR2 (Marx et al., 2000; Wehrens et al., 2006). However, several subsequent experiments by other groups failed to reproduce these findings (Li et al., 2002), turning the attention to serine 2814 (Ser^2814^) site as the primary phosphorylation site responsible for SR Ca^2+^ leak and arrhythmogenic events in HF (Li et al., 2002; Respress et al., 2012). The role of Ser^2808^ phosphorylation was further complicated by the finding that both, minimum and maximum RyR2 phosphorylation at Ser^2808^, increase RyR2 activity, suggesting a U-shaped of RyR2 activity according to the PKA phosphorylation level (Carter et al., 2006). A clear revision of these controversial results is given by Bers (Bers, 2012) and Camors and Valdivia (Camors and Valdivia, 2014).

Ser^2814^ site was described by Wehrens et al. (2004) as a CaMKII site and further evidence confirmed that this site seems to be exclusively phosphorylated by CaMKII. In single-channel experiments, the P_0_ of the RyR2s was generally found to be increased upon phosphorylation by CaMKII (Lokuta et al., 1995; Wehrens et al., 2004; Yang et al., 2007). In line with these results, either activation or overexpression of CaMKII was associated with the positive inotropic effect of β-ARS (Ferrero et al., 2007), an increase of Ca^2+^ spark frequency (Guo et al., 2006) and the susceptibility to arrhythmias (Maier et al., 2003; Dybkova et al., 2011; Respress et al., 2012; Mazzocchi et al., 2016; Valverde et al., 2019). In contrast, animals in which Ser^2814^ was replaced by Alanine (S2814A mice) were protected from arrhythmias and cardiac dysfunction induced by several diseases (van Oort et al., 2010; Di Carlo et al., 2014; Mazzocchi et al., 2016).

Serine 2030 (Ser^2030^) was characterized as a PKA phosphorylation site using classical phospho-epitope mapping (Xiao et al., 2005). Whereas in quiescent cardiac myocytes the RyR2 appears to be completely unphosphorylated (Huke and Bers, 2008), this site has been suggested as the major phosphorylation site in RyR2 responding to PKA activation upon β-ARS in normal and failing hearts (Xiao et al., 2006). In this context, it has recently been described that phosphorylation of RyR2 at Ser^2030^ is required for a complete effect of β-ARS (Potenza et al., 2019) in mouse lines with genetic ablation of this site (RyR2-S2030A).

Interestingly, recent work reports crystal structures of the RyR2 phosphorylation domain with the PKA catalytic subunit (PKAc), showing Ser^2808^ captured within the active site of PKA. The results further demonstrated that the addition of a phosphomimetic at the CaMKII site (S2814D), results in structural changes in the RyR2 phosphorylation domain that enhance the interaction with PKAc. These findings strongly suggest that phosphorylation of Ser^2814^ site may affect the activity of PKA and impact on Ser^2808^, i.e., nearby phosphorylation sites might influence one each other (Haji-Ghassemi et al., 2019). This possible interaction among the different residues sharing the phosphorylation “hotspot” region of RyR2, might clarify previous controversial findings on the role of Ser^2808^ site on different physiological and disease situations. Since RyR2 phosphorylation by PKA and CaMKII may not be independent, the authors suggest that the phosphorylation status of Ser^2808^ may be altered in studies that have used S2814D mice. Of note, in contrast with this prediction, previous experiments indicate that isoproterenol-induced phosphorylation of RyR2-Ser^2808^ site did not vary when isoproterenol was administrated in the absence and presence of a CaMKII inhibitor (KN-93), to avoid the simultaneous phosphorylation of Ser^2814^ residue (Ferrero et al., 2007), i.e., phosphorylation of Ser^2814^ did not influence the extent of phosphorylation of Ser^2808^ site. However, the isoproterenol concentration used in these experiments was rather high and may not allow any further PKA-dependent phosphorylation of this site. Therefore, it would be important to perform similar experiments in the presence of lower isoproterenol concentrations to investigate the possible influence of Ser^2814^ phosphorylation on the isoproterenol-induced phosphorylation of Ser^2808^ site predicted by the crystal structure studies.

The role of phosphatases activity on RyR2 phosphorylation was recently emphasized. It was shown that PP1 activation counteracts the increased kinase activity in human heart failure reducing SR Ca^2+^ leak as well as cellular arrhythmias without significant changes in SR Ca^2+^ load and contractility (Fischer et al., 2018).

Oxidative conditions generally increase the RyR2 P_0_, while reducing agents do the opposite (Marengo et al., 1998; Xu et al., 1998; Salama et al., 2000; Sun et al., 2008). The functional consequence of a moderate cellular oxidative/nitrosative stress could result in an immediate enhancement of Ca^2+^ release from the SR in response to a given physiological trigger. However, severe oxidative stress can cause irreversible and persistent activation of RyR2s (Xu et al., 1998), increasing SR Ca^2+^ leak. It has been reported that NADPH oxidase 2 (NOX2) is the predominant isoform expressed in T-tubules and SR membranes of adult cardiomyocytes. Therefore, it is strategically positioned to modulate the activity of the RyR2s. ROS produced by NOX2 stimulates SR Ca^2+^ release via at least two pathways: direct oxidation or S-glutathionylation of RyR2s or indirectly through CaMKII activation (Palomeque et al., 2009), followed by phosphorylation of the RyR2s. In healthy cardiac muscle neuronal nitric oxide synthase (nNOS) is mainly located in the SR membrane, linked to the RyR2s, which would favor direct RyR2 nitrosation. The role of this PTM of RyR2 on cardiac ECC has been previously reviewed (Lim et al., 2008; Gonzalez et al., 2009).

#### The RyR2 Complex

Ryanodine receptor 2 modulation has been shown to involve also several key proteins (Figure 2B), most importantly CASQ2. CASQ2 not only acts as a Ca^2+^ buffer, but it also mediates the responsiveness of the RyR2 channel to luminal Ca^2+^ by serving as a Ca^2+^ sensor (Gyorke et al., 2004; Gyorke and Terentyev, 2008). This function is effective through protein-protein interactions, with junctin and triadin (Zhang et al., 1997; Shin et al., 2000). However, a direct interaction between CASQ2 and RyR2 has recently been described (Handhle et al., 2016). Junctin is a 26 kDa transmembrane protein forming a complex with triadin, CASQ2, and RyR2. It has been proposed that junctin is in direct contact with RyR2 and works as an anchor for CASQ2 (Zhang et al., 1997; Gyorke et al., 2004).

Calstabin 2 or FKBP12.6, is a peptidyl-prolyl cis/trans-isomerase of 12.6 kDa, associated with RyR2 with a stoichiometry of 4:1 (Figure 2B). The role of FKBP12.6 has been a matter of controversy for over the past two decades (Kaftan et al., 1996; Timerman et al., 1996; Barg et al., 1997; Marks, 2000; Doi et al., 2002; Jiang et al., 2002; Stange et al., 2003; Wehrens et al., 2003; Xiao et al., 2007; Guo et al., 2010). FKBP12.6 has been considered as RyR2 “stabilizer,” since some results indicate that FKBP12.6 dissociation from RyR2 produces RyR2 sub-conductance states and increase the Po of the channel. However, other piece of evidence indicates that FKBP12.6 failed to show any effect of FKBP12.6 on RyR2 gating. The recent identification of FKB12 binding site on RyR2 may help to understand the controversial matters encompassing the FKBP-RyR2 interactions. FKBP12 binds to RyR2 with lower affinity than FKBP12.6, but has a higher cardiac expression level (Jeyakumar et al., 2001). Recent results revealed that only 20% of RyR2 proteins are associated with FKBP12.6 in myocytes (Guo et al., 2010) which could explain why RyR2 is unaffected in FKBP12.6-KO mice (Xiao et al., 2007). Acute overexpression of FKBP12 in adult rabbit ventricular myocytes showed a reduction in the gain of ECC and a decrease in Ca^2+^ spark frequency, suggesting that FKBP12 reduces RyR2 sensitivity to cytosolic Ca^2+^(Seidler et al., 2007). In contrast, more recent results show that FKBP12 activates the RyR2 and competes with FKBP12.6. The last study, would suggest that rather than a direct stabilization of the channel, an increase in FKBP12.6-RyR2 binding competes with FKBP12 at the same binding site and blunts the activation of RyR2 promoted by FKBP12 (Galfre et al., 2012).

Apo-Calmodulin or Ca^2+^-free CaM has an inhibitory effect on RyR2 channel. The Ca^2+^-bound CaM is named Ca^2+^-CaM. Although Ca^2+^-CaM is the usual form that binds to target proteins, CaM can also bind to RyR2. CaM shifts the Ca^2+^-dependence of RyR2 activation to higher Ca^2+^ concentrations (Fruen et al., 2000; Balshaw et al., 2001; Yamaguchi et al., 2005). The role of CaM on RyR2 regulation was highlighted by results that indicate that mutations in CaM are associated with RyR2-mediated cardiac arrhythmias (Nomikos et al., 2014; Sondergaard et al., 2017). High resolution cryo-electron microscopy recently provided new insights into the modulation of RyR2 channel gating by CaM (Gong et al., 2019). These data indicate that Ca^2+^-CaM changes RyR2 conformation differently under different situations. Whereas Ca^2+^-CaM can reverse RyR2 opening by Ca^2+^ and PCB95, a potent channel opener (Samso et al., 2009), it cannot counteract the activation of the channel by a mixture of Ca^2+^, ATP and caffeine. These results emphasize that the P_0_ of RyR2 is critically determined by a strict balance between different activators and inhibitors of the channel (Van Petegem, 2019).

Several proteins that interact with SERCA2a regulating SR Ca^2+^ uptake, also modify the RyR2 function. As already mentioned, the HRC protein is not only associated with SERCA2a but also with triadin. The interaction of HRC with triadin increases with increasing Ca^2+^ concentration (Sacchetto et al., 1999; Arvanitis et al., 2007). This interaction is believed to modulate RyR2 function and SR Ca^2+^ release by conferring refractoriness to SR Ca^2+^ release. In turn, HRC-SERCA2a interaction is also Ca^2+^-dependent. In this case, the maximal HRC-SERCA2a association occurs at low Ca^2+^ concentration and diminishes with increasing Ca^2+^ concentrations (Arvanitis et al., 2007). The different Ca^2+^ dependence of the interaction HRC-SERCA2a and HRC-triadin determines the HRC effects on SR Ca^2+^ handling (Figure 2C): At low SR Ca^2+^, HRC interacts with SERCA2a inhibiting SR Ca^2+^ uptake. When SR Ca^2+^ concentration increases, HRC dissociates from SERCA2a and enhances its binding to triadin, regulating SR Ca^2+^ release (for review, see Arvanitis et al., 2018).

Sorcin is a 22 kDa penta-EF hand Ca^2+^-binding protein expressed in many tissues, including the heart. Single-channel studies indicated that when applied to the cytoplasmic region of RyR2s, sorcin inhibits RyR2 activity in a dose-dependent manner by prolonging the mean close time without modifying single-channel conductance, an effect that is abrogated when sorcin is phosphorylated by PKA. More recent experiments (Farrell et al., 2003) demonstrated that sorcin significantly inhibits both the spontaneous activity of RyR2s in quiescent cells and the Ca^2+^ current (I_Ca_)-triggered activity of RyR2s. Moreover, it decreased the amplitude of the Ca^2+^ transient without affecting the amplitude or kinetics of I_Ca_, reducing the “gain” of ECC mechanism. Sorcin seems to be a key RyR2-associated protein under stress conditions since its ablation displayed a significantly higher incidence of cardiac arrhythmias and sudden death in sorcin-KO mice when subjected to acute or chronic stress challenge (Chen et al., 2018). It has also been shown that sorcin increases SR Ca^2+^ uptake (Matsumoto et al., 2005) and interacts with NCX (Zamparelli et al., 2010) and L-type Ca^2+^ channels (LTCC) (Fowler et al., 2008). All these interactions point to an important role of this protein in ECC regulation.

Also, as in the case of SERCA2a, S100A1 modulates RyR2 function under both diastolic and systolic conditions (Kiewitz et al., 2003; Most et al., 2004; Völkers et al., 2007, Volkers et al., 2010). Most et al. (2004), first demonstrated that addition of S100A1 to isolated SR vesicles resulted in diminished ^3^H-ryanodine ([^3^H]Ry) binding to RyR2 at free Ca^2+^ concentrations of about 150 nM, while a significantly increased [^3^H]Ry binding occurred at Ca^2+^ concentrations greater than 300 nM. Hypothesizing a reduced RyR2 P_0_ at diastolic cytoplasmic Ca^2+^ levels, S100A1 would reduce SR Ca^2+^ leak in quiescent cardiomyocytes (Völkers et al., 2007). Moreover, S100A1 increases fractional SR Ca^2+^ release in voltage-clamped rabbit cardiomyocytes, suggesting that S100A1 enhances the ECC gain under systolic conditions (Kettlewell et al., 2005).

#### Cytosolic and Luminal Ca^2+^ Regulation of RyR2

Both cytosolic and luminal Ca^2+^ regulate RyR2. It has long been known that the release of SR Ca^2+^ in cardiac muscle during ECC is graded by the amount of activating Ca^2+^ outside the SR by the Ca^2+^-induced Ca^2+^ release (CICR) process (Fabiato and Fabiato, 1977). Experimental evidence suggested the presence of high and low affinity Ca^2+^ binding sites in the cytosolic region of RyR2 and luminal Ca^2+^-binding sites, whose luminal occupancy depends on SR Ca^2+^ load (Fabiato and Fabiato, 1979; Shannon et al., 2000). RyR2s are normally closed at low cytosolic Ca^2+^ (100–200 nM); channel activity is maximal at 10–100 μM cytosolic Ca^2+^, while elevating cytosolic Ca^2+^ beyond this point leads to a reduction in P_0_ (Xu and Meissner, 1998). This biphasic behavior implies there are at least two classes of Ca^2+^ binding sites: high-affinity activation and low-affinity inactivation sites. The P_0_ steep dependence of RyR2 on cytoplasmic Ca^2+^, which typically exhibits Hill coefficients of 2–4 (Sitsapesan and Williams, 1994) indicates that RyR2 activation resulted from cooperative involvement of one high-affinity (∼1 μM) Ca^2+^ binding site on each subunit of the homotetrameric channel (Zahradnik et al., 2005). RyR2s also possess two inhibitory sites in their cytoplasmic domains with Ca^2+^ affinities of the order of 1 μM and 1 mM. Mg^2+^ competes with Ca^2+^ at these sites to inhibit RyR2s (for instance, see Laver, 2018).

In 1994, Sitsapesan and Williams were the first to show that luminal Ca^2+^ could directly activate RyR2 (Sitsapesan and Williams, 1994). Since then, different type of evidence supports the notion that luminal Ca^2+^ controls RyR2 function (i.e., Bassani et al., 1995; Gyorke and Gyorke, 1998; Shannon et al., 2000; Kong et al., 2007; Gyorke and Terentyev, 2008). Two different mechanisms have been proposed to explain luminal Ca^2+^ regulation of RyR2: The “feed-through” hypothesis suggests that luminal Ca^2+^ acts on its own cytosolic Ca^2+^ binding site during or after Ca^2+^ passage through an open RyR2 (Herrmann-Frank and Lehmann-Horn, 1996; Xu and Meissner, 1998). A second mechanism proposes that luminal Ca^2+^ regulation of RyR2 is mediated by a Ca^2+^ sensing mechanism inside the SR (Gyorke and Gyorke, 1998; Ching et al., 2000; Jiang et al., 2007; Gyorke and Terentyev, 2008). Although CASQ2, may serve as a key SR luminal Ca^2+^ sensor (Gyorke et al., 2004), experiments in CASQ2-null mice (Knollmann et al., 2006) and in purified native and recombinant RyR2s that lack CASQ2 (Xu and Meissner, 1998; Kong et al., 2007), indicate that RyR2s are also regulated by a luminal Ca^2+^ sensing mechanism that does not require CASQ2. Indeed, a point mutation in RyR2 (RyR2-E4872A) which eliminates Ca^2+^ regulation by luminal but not by cytosolic Ca^2+^ was recently identified (Chen et al., 2014). Structural analysis, at near-atomic resolution, suggests that in addition to E4872, the E4878 residue may also be involved in luminal Ca^2+^ activation of RyR2 (Peng et al., 2016), although the precise mechanism by which each of these different sites promotes luminal Ca^2+^ activation of RyR2 is not clear yet.

## How Can the Disruption of the Normal Interplay Among Sr Ca^2+^ Handling Proteins Evoke Ca^2+^ Triggered Arrhythmias and Apoptosis?

The normal interplay among the different proteins responsible for the release and reuptake of Ca^2+^ by the SR is regulated by different mechanisms as reviewed above. This regulation may be altered and evolve toward different types of cardiac disorders, which include arrhythmias and cell death through apoptotic and necrotic processes. Therefore, regulation and/or alteration of SR Ca^2+^ handling proteins (for instance by phosphorylation, redox changes or mutations), have received great attention from physiologists and clinicians. We will describe below two main consequences of the unbalance of SR Ca^2+^ uptake and release, i.e., Ca^2+^ triggered arrhythmias and cellular apoptosis and necroptosis, with a main focus on those produced by PTM of Ca^2+^ handling proteins.

### Ca^2+^ Triggered Arrhythmias

#### Mechanisms

As stated above, RyR2s are highly regulated molecules. Genetic or PTM of RyR2 are a main cause of Ca^2+^ triggered arrhythmias, i.e., arrhythmias that are originated due to abnormal Ca^2+^ handling.

**FIGURE 3:**
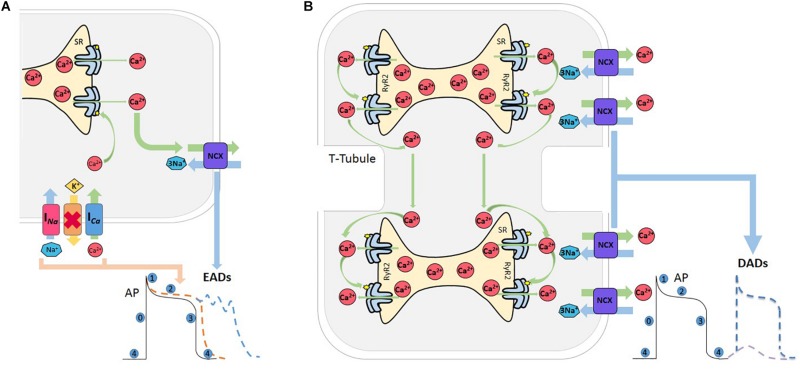
Afterdepolarizations. **(A)** Reactivation of L-type Ca^2+^ current (I_Ca_) during prolongation of the AP, mainly during phase 2 or 3, increases the propensity for EADs. The Na^+^-Ca^2+^ current (NCX) also meets the criterion needed for the positive feedback required for an EAD to occur. Both currents can act synergistically facilitating EAD formation, and thus increasing the probability of an EAD-triggered AP. Red cross indicates inhibition of K^+^ channels. **(B)** Various conditions which increase SR-Ca^2+^ and/or sensitize RyR2 can induce spontaneous Ca^2+^ release from the SR. When this release achieves certain critical mass, Ca^2+^ can induce a saltatory propagation along cell sarcomeres, known as Ca^2+^ waves, which in turn activates the NCX that extrude 2 Ca^2+^ and enter 3 Na^+^, depolarizing the cell membrane and producing a delay after depolarization (DAD). If this depolarization attains the excitability threshold, a spontaneous AP occurs.

Triggered activity describes impulse initiation that is dependent on the so-called afterdepolarizations, which are oscillations in membrane potential that follow the primary depolarization phase (0) of an AP. Afterdepolarizations are divided into early and delayed afterdepolarizations, EAD, and DAD, respectively. EADs are defined as a slowing or reversal of normal repolarization that occurs before completion of AP, usually in phases 2 and 3 of human AP, whereas DADs occur after AP completion (Figure 3). These mechanisms may produce sustained arrhythmias by reentry circuits (Anderson, 2007). EADs occurs usually in the setting of prolonged repolarization and are classically attributed to reactivation of I_Ca_ (January and Riddle, 1989; Nuss et al., 1999). However, a second major current that facilitates EADs formation is NCX. Indeed, experimental evidence indicates that these two currents act synergistically to generate EADs, with their relative contributions varying under specific conditions (Weiss et al., 2010). The late component of the Na^+^ current (I_Na_), has been recognized as an important player to set up the conditions for EADs, by producing SR Ca^2+^ overload, via the reduction of repolarization reserve and the increase in intracellular Na^+^ concentration. In addition, experimental evidence and modeling studies indicate that EADs may directly arise from Na^+^ channel reactivation (Horvath et al., 2013; Sato et al., 2017; Figure 3A).

Delay afterdepolarizations are caused by spontaneous Ca^2+^ releases from the SR (Bers, 2006). Under conditions of SR Ca^2+^ overload and/or in circumstances which sensitize the RyR2s, the Ca^2+^ released by a group of RyR2 activates neighboring RyR2, in such a way that Ca^2+^ propagates in a regenerative way traveling along the myocytes in a saltatory fashion from sarcomere to sarcomere (Cheng et al., 1996). Ca^2+^ waves have substantial arrhythmogenic potential, since they may trigger Ca^2+^ activated currents, such as the NCX current (I_NCX_). This promotes a transient Na^+^ current (It_i_), that depolarizes cell membrane and may eventually trigger a spontaneous AP (Bers, 2006), which is referred to as triggered AP, leading to spontaneous contraction (Spencer and Sham, 2003; Fujiwara et al., 2008; Mazzocchi et al., 2016; Figure 3B).

When referring to a multicellular tissue, e.g., whole heart, spontaneous Ca^2+^ releases synchronized in a small group of cells is not enough for triggering an AP. 3D modeling (Xie et al., 2010) estimated that about 800,000 cells are required to trigger a premature ventricular complex, being able to bring the sink (adjacent tissue in basal conditions) to its activation threshold. A sort of synchronization mechanism must exist for EADs and DADs to overcome the source-sink mismatch. Normally, in an intact tissue, the source-sink mismatch is the main mechanism protecting the heart against spontaneous Ca^2+^ release-induced arrhythmias.

#### The Threshold Concept

**FIGURE 4:**
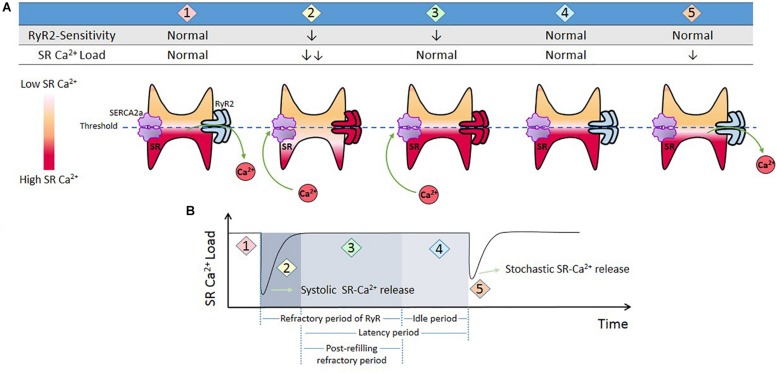
Threshold concept. **(A)** Cartoon that represents different conditions of RyR2 sensitivity and SR-Ca^2+^ load during Ca^2+^ release from SR. When the cell triggers a Ca^2+^ transient (1), SR Ca^2+^ load diminishes and RyR2s enter in a refractory period (2). SR Ca^2+^ uptake produces SR Ca^2+^ recovery (3). During this period there are no spontaneous releases, i.e., RyR2 remained refractory in spite of the fact that SR level has recovered completely (SR *threshold*). This period is followed by what has been named an “idle” period, by Belevych et al., 2012, (4) in which, after SR-Ca^2+^ load and RyR2s recovering, a stochastic activation of RyR2 may trigger a spontaneous release. **(B)** Schematic representation of time dependent changes of SR Ca^2+^ t (1 and 2), and refractoriness (2 and 3) after a stimulus-induced Ca^2+^ transient. (4) “Idle” period during which a spontaneous SR-Ca^2+^ release occurs. We called *post-refilling refractory period* to the period of time needed for refractoriness to recover after SR Ca^2+^ *threshold* was reached.

In the context of Ca^2+^ triggered arrhythmias, an intriguing issue to consider is the SR Ca^2+^ *threshold*. As mentioned above, spontaneous SR Ca^2+^ leak can occur in the absence of membrane depolarization. It has long been known that several conditions that increase SR Ca^2+^ load increase SR Ca^2+^ waves and spontaneous contractions (Orchard et al., 1983; Stern et al., 1983; Wier et al., 1987). Diaz et al. (1997) showed that increasing extracellular Ca^2+^ in quiescent cells produced spontaneous Ca^2+^ release associated with increased SR Ca^2+^ content (Diaz et al., 1997). Once spontaneous Ca^2+^ release arose, further increase in extracellular Ca^2+^ did not affect SR Ca^2+^ content because of the proportional increase in SR Ca^2+^ leak. The authors conclude “It appears there is a maximum level of SR Ca^2+^ content, perhaps because spontaneous release results when the content reaches a threshold”(Diaz et al., 1997). Due to its dependence on the SR Ca^2+^ store, this depolarization–independent SR Ca^2+^ release has been called “Store Overload-Induced Ca^2+^ Release (SOICR)”(Jiang et al., 2004). It has been further shown that the threshold level also depends on the activity of RyR2. One important premise of this mechanism is that “…once a threshold level of SR Ca^2+^ content is reached, SOICR occurs”(Jiang et al., 2004). More recent experiments by Belevych et al. (2012) challenged the idea of *immediacy* that encompasses the last concept. These authors demonstrated that SR Ca^2+^ leak occurs with a substantial time delay after the attainment of diastolic SR Ca^2+^ level, i.e., the attainment of a certain SR Ca^2+^ level is not sufficient for spontaneous Ca^2+^ release and waves generation. The time factor is necessary. Interestingly, the post-refilling refractory period was shorter in myocytes from infarcted hearts than in control myocytes, even though the rate of SR Ca^2+^ content recovery after the stimulus-induced SR Ca^2+^ release was similar. Based on these and other results (Sobie et al., 2005), it was concluded that the probability of spontaneous Ca^2+^ triggering also depends on the recovery of RyR2 from refractoriness (time and Ca^2+^ store–dependent properties of RyR2). In post-infarction myocytes the post-refilling refractory period was reduced, an effect attributed to CaMKII phosphorylation and redox modifications of RyR2. Figure 4 schematizes a possible interpretation of the experimental results.

### Ca^2+^-Induced Apoptosis and Necroptosis

It is generally accepted that mitochondria are at the central stage of cell death (Finkel, 2001; Dorn and Maack, 2013; Pan et al., 2013). Indeed, numerous recent investigations revealed the mitochondria are effectors of programed apoptosis or necrosis and sources of damaging ROS.

Mitochondria are organelle in close association with the SR. This proximity allows a cross-talk between mitochondria and SR which is extremely valuable under normal conditions: Mitochondrial ATP production is crucial for modulation of oxidative phosphorylation and therefore essential to maintain myocyte activity, including SR Ca^2+^ cycling, contraction, and relaxation (Denton, 2009). The physical contact between SR membranes and mitochondria are known as sarco/endoplasmic reticulum (SR/ER)/mitochondria microdomains or mitochondria-associated SR/ER membranes (MAMs). A sufficient local Ca^2+^ concentration may be achieved in specialized microdomains created by the close association of mitochondria and the SR/ER (Szabadkai et al., 2003). In these microdomains, cytosolic Ca^2+^ is predicted to transiently rise to micromolar concentrations, consequently allowing significant Ca^2+^ uptake via the MCU. Physical proximity and functional interplay between mitochondria and SR is maintained in part through tethering of these two organelles by different linkers that may contribute to either decrease or maintain the physical gap between the SR and the mitochondria. For an extensive review see Csordas et al. (2018). On the other hand, perturbation of Ca^2+^ handling may alter mitochondrial-SR Ca^2+^ crosstalk and excessive Ca^2+^ can go to the mitochondria which may contribute to apoptosis and necroptosis in different diseases. Stress conditions that lead to Ca^2+^ or ROS overload trigger mPTP opening, i.e., the mitochondrial membrane becomes permeable to any molecule less than 1.5 kDa in size. Consequent dissipation of the membrane potential (ΔΨm) leads to mitochondrial membrane depolarization, failure to produce ATP and release of mitochondrial proteins such as cytochrome c, which initiate cell death pathways (Bernardi and Di Lisa, 2015). Importantly, it has been shown that mitochondria-initiated cell death is one main mechanism in HF (Nakayama et al., 2007).

### The Interplay Between SR Ca^2+^ Uptake and Leak

As discussed above, the properties of RyR2 are a main factor in determining the magnitude of SR Ca^2+^ leak. However, can an increase in RyR2 P_0_, increase SR Ca^2+^ leak by itself? Experiments by Venetucci et al., clearly demonstrated that the potentiation of RyR2 produces only a transient increase in SR Ca^2+^ leak, because once SR Ca^2+^ leak initiates, SR Ca^2+^ load decreases below the threshold for SR Ca^2+^ leak (Venetucci et al., 2007). Only a simultaneous enhancement of SR Ca^2+^ uptake would be able to maintain the necessary level of SR Ca^2+^ content to attain the threshold for SR Ca^2+^ leak. This conclusion is in agreement with clinical facts showing that patients with catecholaminergic polymorphic ventricular tachycardia (CPVT) due to RyR2 mutation only suffer arrhythmias after β-ARS. Experiments performed in S2814D myocytes, in which Ser^2814^ was mutated to aspartic acid and behaves as pseudo constitutively phosphorylated (van Oort et al., 2010), also showed that an increase in the P_0_ of RyR2 produced by CaMKII phosphorylation was not able to evoke an SR Ca^2+^ leak higher than the one observed in wild type (WT) myocytes unless they are challenged by increasing extracellular Ca^2+^ or β-ARS (Mazzocchi et al., 2016).

**FIGURE 5:**
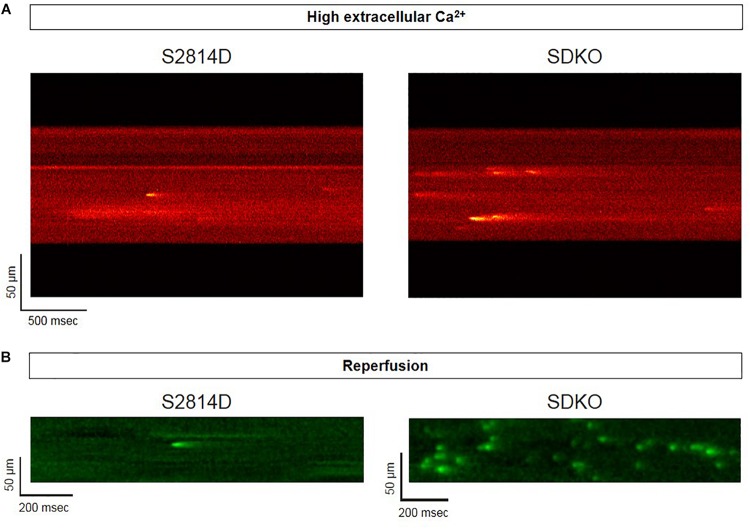
Confocal assessment of spontaneous Ca^2+^ release. **(A)** Representative confocal images showing that Ca^2+^ spark frequency is higher in isolated cardiomyocytes from SDKO mice in comparison to S2814D myocytes (unpublished confocal microscopy records representative of overall data shown in Mazzocchi et al., 2016). **(B)** Similar results were obtained in fluo-4 loaded intact isolated hearts from S2814D and SDKO mice under a confocal microscope, during reperfusion of the hearts submitted to a period of ischemia (unpublished confocal microscopy records representative of overall data shown in Valverde et al., 2019). In both examples, ablation of PLN increases Ca^2+^ spark frequency respect to hearts from S2814D mice.

**FIGURE 6:**
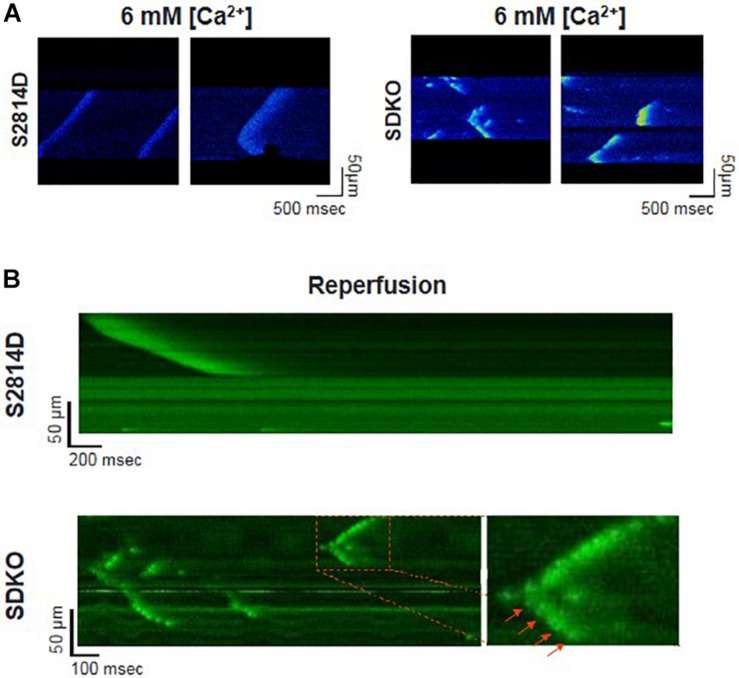
Confocal assessment of spontaneous SR Ca^2+^ waves. **(A)** Representative confocal images showing Ca^2+^ waves in isolated cardiomyocytes from S2814D myocytes (left panel) when exposed to 6 mM extracellular Ca^2+^. When SDKO isolated myocytes are exposed to the same condition, they exhibit miniwaves instead of full propagating Ca^2+^ waves (Reproduced with permission from Mazzocchi et al., 2016). **(B)** Representative confocal images of epicardial Ca^2+^ waves in S2814D and SDKO intact isolated hearts during reperfusion after an ischemic period. SDKO hearts display fragmented Ca^2+^ waves or mini-waves. Red arrows indicate a series of mini-events conserving a Ca^2+^ wave pattern (reproduced with permission from Valverde et al., 2019).

Sarcoplasmic reticulum Ca^2+^ leak is also critically dependent on SR Ca^2+^ load. As stated above, SR Ca^2+^ overload triggers spontaneous Ca^2+^ release via the activation of the RyR2 luminal Ca^2+^ sensor (Gyorke and Terentyev, 2008). Therefore, the potential effect of increasing SR Ca^2+^ load on Ca^2+^ triggered arrhythmias, seems obvious. However, PLN knock-out mice (PLNKO), which have a fully loaded SR, have not proven to be prone to arrhythmias under basal conditions (Santana et al., 1997). Although at first sight these results might suggest that the increase in SR Ca^2+^ load *per se* does not increase arrhythmia propensity, several studies have provided evidence that the increase in SR Ca^2+^ load produced by PLN ablation does produce a dramatic increase SR Ca^2+^ leak (Santana et al., 1997; Huser et al., 1998; Mazzocchi et al., 2016). Unexpectedly, it hardly evokes SR Ca^2+^ waves (Huser et al., 1998; Mazzocchi et al., 2016). Figure 5A shows the results of experiments performed in S2814D mice. As discussed above, this mutation confers the hearts a high propensity to SR Ca^2+^ waves and arrhythmias when submitted to stress (van Oort et al., 2010), and in double mutant mice resulting from cross-breeding S2814D mice with PLNKO mice, to increase SR Ca^2+^ reuptake (SDKO mice) (Mazzocchi et al., 2016; Valverde et al., 2019). In the presence of high extracellular Ca^2+^, the frequency of Ca^2+^ sparks and SR Ca^2+^ leak were higher in SDKO than in S2814D myocytes, consistent with the overall higher SR Ca^2+^ load in SDKO cells (Mazzocchi et al., 2016). Similar results were obtained when SR Ca^2+^ load was increased in both strains subjected to an I/R protocol (Mazzocchi et al., 2016; Valverde et al., 2019; Figure 5B). Unexpectedly, whereas S2814D myocytes displayed full propagating Ca^2+^ waves when exposed to high extracellular Ca^2+^ (Figure 6A) or I/R (Figure 6B), SDKO myocytes mostly show non-propagating Ca^2+^ events, known as mini-waves. Thus, in spite of the higher SR Ca^2+^ leak observed in SDKO myocytes vs. S2814D, the proportion of fully propagating events in SDKO myocytes was significantly less (Mazzocchi et al., 2016). This seeming paradox can be clarified by the acknowledgment that two main factors intervene in the production of arrhythmogenic Ca^2+^ waves: 1. Increased SR Ca^2+^ leak and 2. Cytosolic Ca^2+^ wave propagation.

**FIGURE 7:**
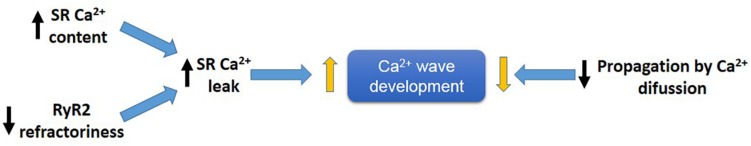
Main factors developing arrhythmogenic Ca^2+^ waves. An increase in SR-Ca^2+^ content and/or a decrease in RyR2 refractoriness favor SR-Ca^2+^ leak and Ca^2+^ wave generation, meanwhile a decrease in propagation of Ca^2+^ by diffusion (e.g., like that produce by PLN ablation), will prevent Ca^2+^ wave propagation by conversion of Ca^2+^ waves into non-propagable Ca^2+^ miniwaves.

An increase in SR Ca^2+^ leak associated with a decrease in propagating SR Ca^2+^ waves indicates a limitation in cytosolic Ca^2+^ diffusion (Figure 7). PLN ablation interrupts cell-wide propagating Ca^2+^ waves, converting them into non-propagated events, like mini-waves or groups of Ca^2+^ sparks (Huser et al., 1998; Mazzocchi et al., 2016; Valverde et al., 2019), supporting the contention that by decreasing cytosolic Ca^2+^, PLN ablation would increase cytosolic Ca^2+^ buffer capacity, hampering Ca^2+^ wave propagation and preventing the arrhythmogenic susceptibility produced by an enhanced SR Ca^2+^ load. Further support to this idea is given by the experiments in which decreasing SR Ca^2+^ uptake by the SERCA2a inhibitor CPA, converts non-propagating mini-waves into full propagating Ca^2+^ waves (Valverde et al., 2019). These results indicate therefore that an increase in SR Ca^2+^ content does increase the propensity to arrhythmias. However, the mechanism by which the increase in SR Ca^2+^ occurred may conspire against the arrhythmogenic effect of the high SR Ca^2+^ load, i.e., an enhancement of SR Ca^2+^ sequestration, if high enough, would increase SR Ca^2+^ load and leak but also preclude Ca^2+^ wave propagation (Huser et al., 1998; Mazzocchi et al., 2016; Valverde et al., 2019).

In contrast, the increase in SR Ca^2+^ leak evoked by increasing SR Ca^2+^ uptake was unable to prevent but rather enhanced heart attack. It was speculated that increasing SR Ca^2+^ uptake was not efficient to hamper the excessive flow of SR Ca^2+^ to the mitochondria, aggravating cardiac damage (Valverde et al., 2019).

### Experimental Evidence

In the following sections, we will give experimental evidence that highlight the importance of Ca^2+^ handling misbalance in the production of Ca^2+^ triggered arrhythmias and cell death. Although the mechanisms of cardiac arrhythmias and apoptosis/necroptosis are usually multifactorial, we will concentrate on experimental examples that emphasize the role of PTM of SR Ca^2+^ handling proteins. When possible, the interplay between Ca^2+^ uptake and release in determining arrhythmias and cardiac damage will be also discussed.

#### Abnormal RyR2 Regulation in the Development of Diabetic Cardiomyopathy

The current typical definition of DCM comprises structural and functional abnormalities of the myocardium in diabetic patients independently of other risk factors, as coronary artery disease or hypertension (Aneja et al., 2008). DCM is the last stage of cardiac damage in Type 1 and 2 Diabetes Mellitus (T1DM and T2DM, respectively) and of metabolic syndrome associated with insulin resistance. The subcellular mechanisms involved in this last stage of DCM have been discussed in several previous reviews (Bugger and Abel, 2014; Cox and Marsh, 2014; Fuentes-Antras, Picatoste et al. 2015, Fuentes-Antras, Picatoste et al. 2015). Most of them concluded that mayor alterations of Ca^2+^ handling, protein expressions, and activities in overt DCM mimic those of HF from different etiologies, including a decrease in SERCA2a activity, SR Ca^2+^ load, systolic Ca^2+^, rate of Ca^2+^ decay, and increase in SR Ca^2+^ leak (Lebeche et al., 2008). Protein glycosylation (Clark et al., 2003) and oxidized CaMKII are significantly up-regulated in the DCM (Jay et al., 2006), therefore CaMKII activation and RyR2 phosphorylation has been proposed as a potential mechanism of heart failure, ventricular arrhythmias and apoptosis in this disease (Daniels et al., 2015). In rats with T2DM, the opening of mPTP in ventricular myocytes was shown to be mainly influenced by the increased ROS and decreased ATP content. It was suggested that Ca^2+^ mishandling due to the slow rate of SR Ca^2+^ uptake could play a role in increasing mPTP opening that might further exacerbate mitochondrial dysfunction and induce cell death (Riojas-Hernandez et al., 2015). Moreover, ROS derived from hyperglycemia trigger myocardial apoptosis by mitochondrial cytochrome c release and consequent activation of the caspase-3 pathway (Cai et al., 2002). Although the underlying mechanism of cell death in DCM is not clear yet, these results suggest that a possible pathway underlying apoptosis in DMC is linked to Ca^2+^ mishandling and mitochondrial-SR Ca^2+^ crosstalk, as described above (For further review about the role of mitochondria on cardiac arrhythmogenesis in DCM see (El Hadi et al., 2019).

**FIGURE 8:**
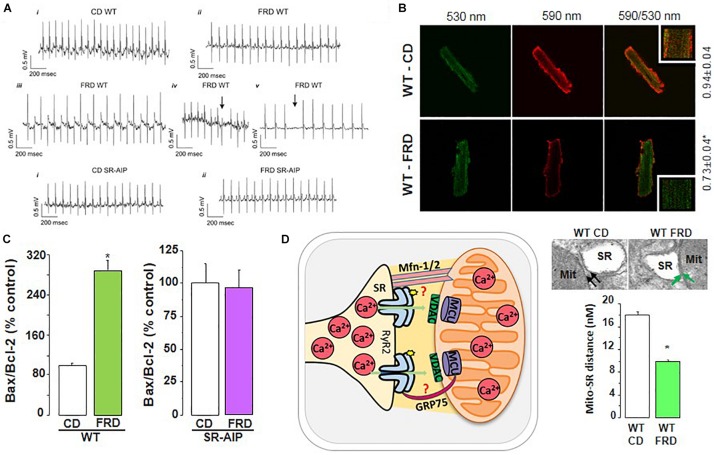
RyR2 dysregulation in DCM development. **(A)** Representative ECG recordings showing that fructose-rich diet (FRD) induces cardiac arrhythmias in WT but not in SR-AIP mice (CD, control diet). WT mice exhibit bradycardia (ii), bidirectional tachycardia (iii), ventricular ectopic beats (iv, arrow), and AV block (v, arrow) when treated with FRD. **(B)** FRD deteriorates mitochondria membrane potential, as shown by an increase in JC-1 green/red fluorescence ratio. **(C)** This alteration in FRD mitochondria is in accordance with a higher apoptotic ratio (Bax/Bcl-2 proteins), which can be prevented in mice expressing the CaMKII inhibitory peptide at the SR level (SR-AIP). **(D)** Histological samples (upper right) show a closer distance between mitochondria and SR in FRD vs. CD treated mice, which might be due to an alteration in structural proteins involved in SR-mitochondria communication. The cartoon in the left side of **(D)** represents this SR-mito interaction (reproduced with permission from Federico et al., 2017). *p < 0.05.

Much less is known about Ca^2+^ handling and mishandling and the potential occurrence of arrhythmias and apoptosis in the first stages of this disease, in which subclinical events develop for years before the clear emergence of HF symptoms. A possible cause for this lack of information may lie in the poor diagnosis of this stage of the disease and in the different models used to study prediabetic molecular events (King, 2012; Graham and Schuurman, 2015; King and Bowe, 2016). Indeed, the results that reveal the underlying mechanisms of the pathogenesis of DCM, are different according to the model, the degree of evolution of the disease before reaching DCM and the gender explored. Examples of conflicting results at the level of Ca^2+^ handling and particularly of RyR2 are observed in the metabolic syndrome model, (db/db mice), which lacks leptin receptors (Chen et al., 1996). In male db/db mouse hearts, the levels of RyR2 were found to be depressed and the RyR2 phosphorylation at the CaMKII site was not altered. However, RyR2 phosphorylation at the PKA site was found to be increased (Pereira et al., 2006, 2014). Intriguingly, db/db female mouse hearts showed no changes in RyR2 expression associated with a decrease in PKA and CaMKII RyR2 phosphorylation sites (Pereira et al., 2014). In a model of fructose-rich diet (FRD) applied to male rats and mice, our laboratory has described that this hypercaloric diet, which also induces insulin resistance, increases CaMKII activation and RyR2 dysfunction due to Ser^2814^ phosphorylation (Sommese et al., 2016; Federico et al., 2017). On the other hand, experiments *in vitro* revealed that during acute hyperglycemia, RyR2 activity can also be altered. Hyperglycemia leads to *O*-Glc-NAcylation of proteins such as CaMKII. Erickson et al. elegantly showed that the acute increase of glucose or *O*-linked N-acetylglucosamine is directly responsible for CaMKII-dependent diastolic RyR2 Ca^2+^ leak and SR Ca^2+^ load depletion in hyperglycemia (Erickson et al., 2013). Additionally, we recently demonstrated that in an early diabetic stage, prevention of CaMKII activation by ROS avoided SR Ca^2+^ leak evoked by CaMKII-dependent phosphorylation of RyR2 (Sommese et al., 2016). Thus, the available results indicate that a main disturbance of Ca^2+^ handling in prediabetic hearts occurs at the level of RyR2 phosphorylation with the consequent increase in SR Ca^2+^ leak and the possibility of triggered arrhythmias and cell death. Indeed, during prediabetic states, the risk of cardiovascular events is already increased and myocardial abnormalities might appear before the diagnosis of T2DM. These alterations are thought to root irregularities at the cardiac myocyte level, ultimately contributing to structural and functional anomalies observed in DCM (Hajat et al., 2004). Actually, in the mouse model of impaired glucose tolerance (IGT) mentioned above (FRD-induced insulin resistance), we found serious cardiac disorders (Sommese et al., 2016; Federico et al., 2017). The fact that this model lacks the more frequent co-morbidities of DCM, supports the metabolic origin of the alterations at the cell level. These animals develop remarkable cardiac remodeling (Sommese et al., 2016). Also, ventricular myocytes exhibit cardiac arrhythmogenic events leading to ventricular arrhythmias which can be prevented in transgenic mice expressing the CaMKII inhibitor AIP targeted to the SR membranes, avoiding phosphorylation of SR proteins (PLN and RyR2) by CaMKII (SR-AIP mice) (Figure 8). Moreover, a ROS scavenger as tempol could avoid RyR2 phosphorylation and SR Ca^2+^ leak, preventing the arrhythmogenic pattern of the prediabetic cells (Sommese et al., 2016). Of note, the increase in RyR2 phosphorylation observed in FRD myocytes decreases SR Ca^2+^ content. This decrease occurs in spite of the increase in SERCA2a activity which contributes to preserving SR Ca^2+^ load. This increase, which may be due to the CaMKII-dependent increase in Thr^17^ phosphorylation of PLN, would contribute to avoiding a further decrease in SR Ca^2+^ load but would also favor SR Ca^2+^ leak and arrhythmogenic Ca^2+^ waves.

Apoptosis is also an early sign of myocardial dysfunction in the evolution of the diabetic disease, preceding the increase in collagen which may lead to structural and irreversible alterations (Federico et al., 2017). We described a cascade of events initiated by a CaMKII-induced increase in SR Ca^2+^ leak which is linked to mitochondrial membrane depolarization and cardiac damage. A particularly striking finding was the CaMKII-induced remodeling of SR-mitochondria microdomains. The latter would strongly support SR–mitochondria dialogue, facilitating Ca^2+^ drain to the mitochondria and cell death, in the scenario of an increased SR Ca^2+^ leak (Federico et al., 2017). The elucidation of the intracellular signaling pathway of this altered SR-mitochondria relationship would further contribute to the knowledge of DCM molecular alterations. Further investigations are needed to examine the proteins involved in the SR-mitochondria communication (like mitofusin-2, Mfn2, and the chaperone glucose-related protein 75, GRP-75), a completely unexplored field in this disease.

#### Ca^2+^ Triggered Arrhythmias Induced by Digitalis Intoxication

Cardiac glycosides have been used for the treatment of HF over the last 200 years due to their inotropic properties (Altamirano et al., 2006; Gonano et al., 2011). Although many doubts about their safety in HF treatment have emerged mainly at the end of the last millennium, it is still considered a valuable cardiac tool in some particular scenarios (see for review Whayne, 2018). Unfortunately, these compounds have a very narrow therapeutic range due to their toxic effects that include an enhanced propensity to arrhythmias. The arrhythmic effects of cardiac glycosides have been traditionally attributed to an increase in SR Ca^2+^ load which, by leading to an increase in Ca^2+^ leak, would evoke cytosolic Ca^2+^ waves and triggered arrhythmias (Wier and Hess, 1984; Fujiwara et al., 2008; Eisner et al., 2009; Weiss et al., 2011). More recent experiments indicated that a change in RyR2 may be also involved in cardiac glycosides–induced arrhythmias. Experiments by Gyorke’s group (Ho et al., 2011) indicate that the arrhythmogenic effect of cardiotonic glycosides is linked to NOX2-dependent ROS release from mitochondria. The increase in ROS was initially thought to produce RyR2 thiol oxidation that would increase the sensitivity of the channel to luminal Ca^2+^, thus lowering the critical SR Ca^2+^ content at which spontaneous Ca^2+^ waves occur (Terentyev et al., 2008). However, simultaneous experiments by Gonano et al., indicated that ouabain-induced arrhythmias requires CaMKII activation: Chronic administration or high–toxic doses of ouabain administered acutely, increased CaMKII activity in mouse hearts (Gonano et al., 2011). Moreover, inhibition of CaMKII was able to prevent spontaneous contractions in isolated myocytes and arrhythmias in intact mouse hearts, without affecting ouabain inotropic action. These experiments also showed that CaMKII phosphorylates both, RyR2 and PLN, which would increase SR Ca^2+^ leak and SERCA2a activity. Although this later effect would add to the increase in SR Ca^2+^ load resulting from Na^+^-K^+^-ATPase inhibition, these experiments concluded that CaMKII-dependent PLN phosphorylation might not contribute to ouabain–induced increase in SR Ca^2+^ content and inotropic effect since they were of similar magnitude in the absence and presence of CaMKII inhibition. However, since CaMKII dependent phosphorylation of RyR2 was also inhibited, it might be that the resultant similar SR Ca^2+^ load observed after CaMKII inhibition was due to the prevention of SR Ca^2+^ leak. Moreover, the fact that CaMKII inhibition prevents arrhythmias without affecting the ouabain-induced increase in SR Ca^2+^ load would suggest that the increase in SR Ca^2+^ content produced by the drug is not enough to reach the necessary *threshold* to trigger, by itself, ouabain-induced arrhythmias (Gonano et al., 2011). An increase in RyR2 sensitivity is needed.

The role of CaMKII-dependent induced increase in SR Ca^2+^ leak and ventricular arrhythmias was later confirmed by experiments by Gyorke’s group, which revealed that replacement of Ser^2814^ site of RyR2 by Ala -a non-phosphorylatable amino acid-prevents ouabain-induced Ca^2+^ leak and arrhythmias. These results definitively confirmed that phosphorylation, rather than RyR2 oxidation, was required for the increase in channel spontaneous activity and arrhythmogenesis in the context of digitalis toxicity. Instead, the increase in ROS would contribute to CaMKII activation that in turn produces the observed RyR2 phosphorylation (Palomeque et al., 2009; Ho et al., 2014).

Importantly, other studies have shown that mitochondria are also involved in the toxic and arrhythmogenic effects of cardiotonic glycosides. The results of these investigations indicate that ouabain-induced increase in cytoplasmic Na^+^ compromises mitochondrial energetics and redox balance by blunting mitochondrial Ca^2+^ accumulation. Improving mitochondrial Ca^2+^ retention by inhibition of mitochondrial NCX, can mitigate these effects, suppress Ca^2+^-triggered arrhythmias and improve the positive inotropic effects of cardiac glycosides (Liu et al., 2010).

#### Abnormal Ca^2+^ Handling in Ischemia/Reperfusion

Ischemic heart disease is a leading cause of mortality worldwide. Cardiac ischemia reduces cardiac output and promotes arrhythmias and cell death. Reperfusion therapies are the standard treatment for patients suffering myocardial infarction, however, re-establishing blood flow is associated with additional cell damage (I/R injury), and exacerbating the effect of the preceding ischemia. Indeed, it was shown that reperfusion may trigger life-threatening arrhythmias, accounting for up to half of the total I/R-induced infarcts (Braunwald and Kloner, 1985; Yellon and Hausenloy, 2007; Said et al., 2011; Garcia-Dorado et al., 2012). Although the factors contributing to I/R injury are complex (see for review (Murphy and Steenbergen, 2008). I/R injury constitutes another example in which experimental evidence reveals that disturbed Ca^2+^ handling and mitochondria ROS production are main responsible for reperfusion arrhythmias and cardiac damage (Valverde et al., 2006, 2010; Mattiazzi et al., 2015; Bagheri et al., 2016). The increase in cytosolic Ca^2+^ during ischemia was associated with an enhancement of SR Ca^2+^ load (Valverde et al., 2010), which is released at the onset of reperfusion and produces an abrupt rise in cytosolic Ca^2+^ (Ca^2+^ *bump*) and the consequent decrease in SR Ca^2+^ content and Ca^2+^ transient. Moreover, a major mechanism for the ischemia-induced increase in diastolic Ca^2+^ is an increase in the frequency of Ca^2+^ sparks which may switch to arrhythmogenic Ca^2+^ waves during reperfusion (Mattiazzi et al., 2015).

Reactive oxygen species/reactive nitrogen species are generated during reperfusion by several different cellular sources, being the mitochondria the more important one. Mitochondrial Ca^2+^ overload and subsequently ROS production trigger mitochondrial permeability transition pore and ROS production via ROS-induced ROS release mechanisms (Zorov et al., 2000). Both, Ca^2+^ mishandling and ROS production set an ideal intracellular milieu for activation of CaMKII, which play a main role in I/R arrhythmias, apoptosis, and necroptosis. *Ex vivo* and *in vivo* experiments described an increase in phosphorylated-CaMKII (p-CaMKII) and oxidized-CaMKII (ox-CaMKII) at the onset of reperfusion (Said et al., 2011; Becerra et al., 2016), which was associated to a significant increase in the phosphorylation of Thr^17^ site and RyR2 Ser^2814^ (Vittone et al., 2002; Salas et al., 2010; Ling et al., 2013), as well as redox changes of RyR2 (Becerra et al., 2016). Reperfusion arrhythmias are largely dependent on SR Ca^2+^ leak evoked by these PTM of RyR2 (Said et al., 2008; Becerra et al., 2016). In this scenario the role played by a substantial increase in SR Ca^2+^ uptake was similar to that observed in stress-induced Ca^2+^-triggered arrhythmias, i.e., increasing SR Ca^2+^ uptake by PLN ablation protects against reperfusion arrhythmias. This protection was achieved by alteration of Ca^2+^ wave propagation, which were transformed in non-arrhythmogenic mini-waves and reconverted in full Ca^2+^ waves in the presence of SERCA2a inhibition (Figure 6C; Valverde et al., 2019). Of note, PLN ablation is equivalent to a situation of permanent maximal PLN phosphorylation as stated above. In ischemic reperfused WT hearts, PLN phosphorylation is highly but transiently increased at the onset of reperfusion (Vittone et al., 2002; Said et al., 2008). Under these conditions, we did not observe Ca^2+^ mini-waves (Valverde et al., 2010). This would mean that Thr^17^ phosphorylation at the onset of reperfusion is unable to prevent SR Ca^2+^ waves progression. In contrast, it might help to increase SR Ca^2+^ load and maintain SR Ca^2+^ leak. This contention is supported by experiments in which hearts were subjected to a short I/R protocol to produce stunning. In the stunned heart, the transient phosphorylation of Thr^17^ of PLN is essential for contractile recovery upon reperfusion, even though phosphorylation of RyR2 also occurs and induces reperfusion arrhythmias (Said et al., 2003, 2011).

After a prolonged ischemic period, reperfusion evokes irreversible cardiac injury. Under these conditions, myocytes die by apoptosis, autophagy, and necrosis. The rise in Ca^2+^ during I/R leads to mitochondrial Ca^2+^ accumulation, which is greatly favored by the close association between mitochondria and SR and constitutes a main event in the initiation of cell death (Rizzuto and Pozzan, 2006). Experimental evidence consistently indicates that CaMKII inhibition is protective in the irreversible I/R injury (Zhang et al., 2005; Vila-Petroff et al., 2007; Salas et al., 2010). Although the mechanisms for myocardial protection by CaMKII inhibition are still unclear, one of the CaMKII deleterious pathway in I/R certainly involves the SR and the mitochondria (Vila-Petroff et al., 2007; Salas et al., 2010; Valverde et al., 2010; Joiner et al., 2012). In previous experiments, we showed that I/R damage was diminished in hearts from S2814A mice. Conversely, in the hearts of S2814D mice (constitutively pseudo-phosphorylated), cardiac damage increased (Di Carlo et al., 2014). A decrease in the expression of RyR2 described in I/R (Salas et al., 2010), compatible with a degradation/damage of these channels (Pedrozo et al., 2010) and changes in RyR2 activity induced by redox alterations, may contribute to increase SR Ca^2+^ leak (Hidalgo et al., 2004; Said et al., 2011; Di Carlo et al., 2014). These alterations in RyR2 would add to the deleterious action of RyR2 phosphorylation favoring the increase in mitochondria Ca^2+^ content and greatly contributing to necroptosis and apoptosis in reperfusion cardiac damage (Salas et al., 2010; Di Carlo et al., 2014). This cascade would be further stimulated by the increase in CaMKII-dependent phosphorylation of MCU described by Joiner, Koval et al.(Joiner et al., 2012). However, recent experiments do not support a relevant role of CaMKII for mitochondrial Ca^2+^ uptake in cardiac myocytes at least under physiological conditions (Nickel et al., 2019).

As stated above, the transient increase in CaMKII-dependent PLN phosphorylation plays a beneficial role in the stunned heart. The role of PLN phosphorylation in I/R injury is less clear. After prolonged ischemia, we showed that preventing PLN phosphorylation exacerbates the functional and structural heart damage after myocardial infarction, suggesting that CaMKII-dependent phosphorylation of PLN observed during reperfusion favors post-ischemic recovery and protects from I/R cardiac damage (Di Carlo et al., 2014). However, the fact that I/R does produce cardiac damage indicates that even being beneficial, CaMKII-dependent PLN phosphorylation results insufficient to counteract the effect of simultaneous detrimental mechanisms that take place during I/R. On the other hand, several groups have tested the effects of increasing SR Ca^2+^ uptake on cardiac damage during I/R injury, by different maneuvers. The outcome of these experiments is controversial. For instance, Yang et al. (2006) demonstrated that the protective effect of chronic CaMKII inhibition in AC3I mice was lost when they were interbred with PLNKO mice and submitted to myocardial infarction, supporting a detrimental effect of enhancing of SR Ca^2+^ uptake. Similar results were obtained in our laboratory. The ablation of PLN in SDKO mice increases Ca^2+^ leak upon reperfusion (Figure 6A). This increase was associated with an increase in infarct size and mitochondrial dysfunction. Therefore, these experiments demonstrated that an important increase in SR Ca^2+^ uptake as that produced by PLN ablation, was able to prevent reperfusion arrhythmias, but failed to prevent, and even enhance, cardiac damage (Valverde et al., 2019). The important increase in SR Ca^2+^ uptake would favor the unbalance between SR Ca^2+^ uptake and leak, promoting mitochondrial Ca^2+^ overload and cell death. Other studies demonstrated that accelerating SR Ca^2+^ uptake by different means (i.e., overexpressing SERCA1a, with higher kinetics than SERCA2a, or expressing a repressor of PLN activity, PP1 H-1), alleviated post-ischemic cardiac injury (Talukder et al., 2007, 2008; Nicolaou et al., 2009a), supporting a beneficial effect of accelerating SR Ca^2+^ uptake. These controversial results seem not to arise from species differences since most of the experiments mentioned above referred to rodents. It is possible that the final beneficial or detrimental outcome of increasing SR Ca^2+^ uptake might tightly depend on the extent of Ca^2+^ uptake and SR Ca^2+^ load achieved during ischemia and at the onset of reperfusion. For instance, moderate increases in SR Ca^2+^ content have been associated with beneficial effects (Nicolaou et al., 2009b), whereas more important increases, like those expected in PLNKO mice, were associated with detrimental actions (Valverde et al., 2019).

### Is RyR2 Activation Always Detrimental?

We have previously associated the increase in RyR2 activation, for instance by CaMKII-dependent phosphorylation, with cardiac damage and arrhythmias due to exacerbated diastolic SR Ca^2+^ leak, as discussed above. However, it is important to bear in mind that potentiation of RyR2 activity persists during systole and enhances systolic fractional Ca^2+^ release, bringing the heart to a new state of higher efficiency. This would allow the heart to maintain a given contractility despite a decrease in SR Ca^2+^ content or to enhance contractility if SR Ca^2+^ content is simultaneously preserved (for further discussion, see Lascano et al., 2017).

## Concluding Remarks

The normal interplay among the proteins involved in SR Ca^2+^ uptake and release is a main determinant of the regular beat to beat contractile function of cardiac myocytes. Regulation and deregulation of these proteins are crucial to understanding the balance between SR Ca^2+^ uptake and leak, responsible for SR Ca^2+^ content and myocardial contractility, as well as its unbalance, which determines an excess of SR Ca^2+^ leak, able to produce arrhythmias and cardiac damage. Post-translational enhancements of SR Ca^2+^ uptake have a beneficial effect resulting in a detectable increase in contractility when the unbalance between SR Ca^2+^ uptake and leak favors the uptake. This is the case of PLNKO mice and stunned hearts. When SR Ca^2+^ leak is increased by the enhancement of RyR2 activity, the increase in SR Ca^2+^ uptake may not be enough to counteract SR Ca^2+^ leak, resulting in two opposite effects: (a) beneficial, by opposing to SR Ca^2+^ leak and rescuing at least in part SR Ca^2+^ load and contractility; (b) detrimental, because the increase in SR Ca^2+^ load would favor SR Ca^2+^ leak, arrhythmias, and cardiac damage. Interestingly, a greatly exacerbated increase in SR Ca^2+^ uptake, as that produced by PLN ablation, does contribute to increasing SR Ca^2+^ leak and cardiac damage by incrementing SR Ca^2+^ load and leak. Paradoxically, and in spite of the exacerbated SR Ca^2+^ leak, this increase may prevent Ca^2+^ triggered arrhythmias, by a different mechanism, i.e., diminishing cytosolic Ca^2+^ and avoiding Ca^2+^ wave propagation. Post-translational activation of RyR2 activity would produce a deleterious effect by increasing SR Ca^2+^ leak and predisposing to cardiac damage and arrhythmias. When this modification occurs only at the level of SR Ca^2+^ release/leak, the short-lived enhancement of SR Ca^2+^ leak may produce a decrease in contractility due to the decrease in SR Ca^2+^ load, without any further detrimental effect. Moreover, the increased activity of RyR2 during systole tends to preserve contractility, even at lower SR Ca^2+^ loads. The detrimental effect of RyR2 activation (i.e., arrhythmias and cardiac damage) can only take place when it occurs associated to an increase in SR Ca^2+^ uptake, able to maintain SR Ca^2+^ leak. This is the typical case of Ser2814D myocytes, with constitutive pseudo-phosphorylation of RyR2 at the CaMKII site.

## Author Contributions

JP and AM generated the idea. JP, AM, MF, and CV wrote the manuscript, designed, and edited the figures.

## Conflict of Interest

The authors declare that the research was conducted in the absence of any commercial or financial relationships that could be construed as a potential conflict of interest.

## Abbreviations

β-ARS: β-adrenergic stimulation
AGEs: advanced glycation end products
AP: action potential
Ca^2+^: calcium
CaM: calmodulin
CaMKII: Ca^2+^-calmodulin dependent Kinase II
CASQ2: calsequestrin
CPA: cyclopiazonic acid
DAD: delay afterdepolarizations
DCM: diabetic cardiomyopathy
EAD: early afterdepolarizations
ECC: excitation contraction coupling
ER: endoplasmic reticulum
HRC: histidine-rich Ca^2+^binding protein
I/R: ischemia/reperfusion
MCU: mitochondrial Ca^2+^ uniporter
Na^+^: sodium
NCX: Na^+^/Ca^2+^ exchanger
NO: nitric oxide
ONOO^–^: peroxynitrite
P_0_: open probability
PKA: protein kinase A
PLN: phospholamban
PP1: type 1 phosphatase
PTM: post-translational modification
RNS: reactive nitrogen species
ROS: reactive oxygen species
RyR2: ryanodine receptor 2
SERCA2a: sarcoplasmic reticulum Ca^2+^-ATPase
SR: sarcoplasmic reticulum
SUMO1: small ubiquitin-like modifier type 1
T2DM: type 2 diabetes mellitus.
